# The Use of Barley Malt as a Binder in Molding Sand Technology

**DOI:** 10.3390/ma15093375

**Published:** 2022-05-08

**Authors:** Bartłomiej Samociuk, Daniel Medyński, Daniel Nowak, Joanna Kawa-Rygielska, Kacper Świechowski, Alan Gasiński, Andrzej Janus

**Affiliations:** 1 Department of Light Element Engineering, Foundry and Automation, Wroclaw University of Science and Technology, Wybrzeże Wyspiańskiego 27, 50-370 Wroclaw, Poland; bartlomiej.samociuk@pwr.edu.pl (B.S.); daniel.nowak@pwr.edu.pl (D.N.); 2 Faculty of Technical and Economic Sciences, Witelon Collegium State University, Sejmowa 5A, 59-220 Legnica, Poland; andrzej.janus@pwr.edu.pl; 3 Department of Fermentation and Cereals Technology, Wroclaw University of Environmental and Life Sciences, pl. Grunwaldzki 24A, 50-363 Wroclaw, Poland; joanna.kawa-rygielska@upwr.edu.pl (J.K.-R.); alan.gasinski@upwr.edu.pl (A.G.); 4 Department of Applied Bioeconomy, Wroclaw University of Environmental and Life Sciences, Chełmońskiego 37A, 51-630 Wrocław, Poland; kacper.swiechowski@upwr.edu.pl

**Keywords:** casting alloys, molding sands, casting binders, quality of castings

## Abstract

The aim of this study was to attempt to use barley malt as a natural, organic binder in the technology of molding sand. TGA analysis of the binder was performed, during which temperatures of thermal decomposition of its components were determined. The results of TG/DTG analysis show that a loss of ~75% of mass of the MB binder is organic matter. Over 50% of this is starch. The results indicate the possibility of using a binder made of barley malt as a binding material for quartz sand grains. This fact was confirmed by tests carried out with use of SEM. During the observations, it was found that barley malt forms smooth bridges connecting individual grains of quartz sand. The typical properties of molding sands with barley malt were also determined, compared to sands containing commonly used binders. At the same time, the influence of the content of this binder on flowability, permeability, strength properties, and wear resistance was assessed. It has been found that increasing the binder content in molding mass results in an increase in strength and wear resistance, as opposed to flowability and permeability. Test castings were also made. It was found that the addition of a binder made of barley malt has a positive effect on the surface quality of castings. This was confirmed by roughness measurements of the test castings. At the same time, a tendency to excessive gas evolution during pouring was shown, with higher contents of this binder. Moreover, greater amounts of barley malt in the molding sand (MB 5%) as compared to the lower content (MB 2%) increased the thickness of the burnt layer of the sand by 25%. This is due to the exothermic reaction when more binder is burnt. It is extremely important from the point of view of the regeneration of molding sand.

## 1. Introduction

The foundry industry has been looking for new solutions aimed at improving the quality of finished products for a long time. The introduction of new technologies and materials was usually associated with an increased risk of factors harmful to people and the environment. Employees employed directly in the foundry process have for many years belonged to the group with an increased occupational risk. Molding sands used during the pouring of molds with liquid metal are a source of emission of harmful, toxic, and carcinogenic substances (benzene, PAH). This is mainly caused by use of synthetic binders, such as, e.g., phenol-formaldehyde, urea-formaldehyde, furan, and furfuryl resins [1,2].

However, for several decades, the guidelines of international bodies have forced manufacturers to seek solutions that are friendly to humans and the environment. The new guidelines for the foundry industry limit the use of harmful, toxic materials that causethe emission of hazardous gases, noise, and vibrations, as well as those that cannot be reused after the recycling process [3,4,5]. As a result, these activities led to the emergence of research on the use of new or previously unused molding materials.

In the last decade, research has appeared on the possibility of replacing synthetic binders with materials of plant origin [6,7,8]. Literature reports indicate the possibility of using materials such as wood rosin, molasses, oils, dextrin, starch, cellulose, and natural latex [9,10,11,12]. The presented materials may be an intrinsic binder shaping the properties of molding sand or a special additive influencing selected properties of the sand. This is carried out in such a way as to meet the requirements of the foundry and to meet the new environmental protection and health and safety regulations [3].

When comparing masses based on barley malt binders with other masses made with organic binders of vegetable origin, it can be seen that the mass with malt has better or comparable permeability. Depending on the binder content, its permeability varies between 317–240 × 10^−8^ m^2^/Pa∙s. On the other hand, the mass with a cassava binder (6%) obtained the result of 126 × 10^−8^ m^2^/Pa∙s, and the mass with rice starch 122–341 × 10^−8^ m^2^/Pa∙s and corn starch 156–350 × 10^−8^ m^2^/Pa∙s [6,13]. On the other hand, the compressive strength is definitely better, as it is above 5 MPa, compared to the mass with addition of: cassava—0.51 MPa; rice—0.065 MPa; corn—0.050 MPa [6,13]. Other parameters were not compared due to the lack of data.

Therefore, authors of the work undertook research attempts aimed at assessing the possibility of using barley malt as a binder for molding and core sand, in relation to typical sand. The fact that malt is an organic material, derived from renewable sources and with a low environmental impact, is why it fits in the current trend of sustainable development.

## 2. Materials and Methods

### 2.1. Determination of Starch Content in Barley Malt

For the research, malt in form of dried and finely ground malted barley flour was used, similarly to the use of other organic materials used as a binder in the technology of molding sand [14,15]. From a chemical point of view, malt consists of carbohydrates (especially starch, sugars, and dextrins), protein compounds (amylolytic, proteolytic, and cytolytic enzymes) and small amounts of fatty compounds [16].

The determination of starch in barley malt as a binder for molding sand was performed with the polarimetric method using an AA-55 polarimeter (Optical Activity Ltd., Huntingdon, UK). This method consists in dissolving the malt in diluted hydrochloric acid and, after clarifying the solution, measuring the twist of the plane of polarized light. Starch content was calculated from the Biot formula, assuming that the specific rotation of starch dissolved in HCl is 183.7°. Marking was made in accordance with PN-EN-ISO 10520: 2002—Native starch—Determination of starch content—Ewers polarimetric method [17,18].

### 2.2. Determination of Protein Content in Barley Malt

The total amount of protein in barley malt was determined using the Kjeldahl method using the Kjeltec 8100 analyzer (Foss, Copenhagen, Denmark) [19,20]. This method consists in the mineralization of the sample in a medium of concentrated sulfuric acid (VI) in the presence of catalysts. Protein nitrogen was converted under these conditions to the ammonium ion which, after alkalinization, was distilled in the form of ammonia. The ammonia was determined by acid-base titration with 0.01 MHCl. The standard PN-EN-ISO 20483: 2014-02 was used for marking—Cereals and pulses—Determination of the nitrogen content and calculation of the crude protein content—Kjeldahl method.

### 2.3. Thermogravimetric Analysis of Barley Malt

Thermogravimetric analysis (TGA or TG) is a method on the basis of which it is possible to assess the temperature at which the test sample decomposes, the loss of binding properties, and burning. The authors proved that thermal analysis can be used in research on casting materials—in this case, the binder of barley malt [21,22,23,24,25,26]. Tests were carried out using the RST 40 × 200/100 tube furnace (Czylok, Jastrzębie-Zdrój, Poland) connected to the PS 750.3Y analytical balance (Radwag, Radom, Poland).

Samples weighing approx. 2 g were heated to 850 °C at three different rates: 12.5 °C/min, 25 °C/min, 50 °C/min. Changes in samples masses were recorded at one-second intervals with an accuracy of 0.001 g.

The obtained TGA curves were smoothed (with a scatter parameter of 0.1) by the local estimated scatter plot smoothing (LOESS) method using the OriginPro 2019b software (OriginLab, Northampton, MA, USA). Then, on the basis of the smoothed TG plot, the DTG derivative was calculated dTG/dT, where dTG (%) is a change in sample mass and dT (°C) a change in its temperature. In this way, the change in mass of the sample was determined, with the change in its temperature by 1 °C.

### 2.4. Preparation of Molding Sand

Molding sands were prepared according to the instructions in the literature [3]. Preparation of the masses began with mixing dry ingredients together. Dry mixing time was one minute. For this purpose, the LM-1 laboratory roller mixer (Multiserw-Morek, Marcyporęba, Poland) was used. Then distilled water was added and all the ingredients were mixed for another 3 min. For all prepared mixes, the ambient conditions were the same, i.e., a temperature of 21 °C and an air humidity of about 40%. After mixing, the molding sand was stored for 60 min in a tightly closed container. The total mass of the dry ingredients was 5 kg.

Five molding sands based on quartz sand from Grudzeń Las mine, with a main fraction of 1K class 0.20/0.16/0.10, were tested, in accordance with the requirements of PN-85/H-11001. The composition of the prepared molding sands is presented in Table 1.

Two masses contained the addition of barley malt binder in the amount of 2% (MB 2%) and 5% (MB 5%) and water (2% and 5%, respectively). The third mass was made with 5% sodium water glass (WG 5%). The fourth mass (B 8%) contained Specjal bentonite in an amount of 8% and 0.8 parts by weight of water. On the other hand, the fifth thermosetting mass (RCS) was factory-prepared by Zębiec from quartz sand with the same main fraction as the other four masses. The binder of this mass was novolak resin. The commercial designation of the sand was ZGM D0128.

The results of all the tests performed were the average value obtained from at least three measurements, taking into account the permissible measurement error.

### 2.5. Preparation of Samples for Testing the Properties of Molding Sand

The preparation of samples for determining the properties of molding sands consisted in making three standard types of measuring samples: cylindrical, elongated, and octal (dog-bone) [3]. The samples were compacted on an LM-1 standard (Multiserw-Morek, Marcyporęba, Poland) hand compactor. It consisted in hitting the mass in a specific modeling block with a rammer three times. The compaction work was about 9.8 J. Then samples were dried in a laboratory dryer SLW 115 (Pol-Eko-Aparatura, Wodzislaw Śląski, Poland) with forced air circulation at the temperature of 150 °C for 60 min.

### 2.6. Methods of Determining the Properties of Molding Sand

In order to determine the properties of molding sands, tests were carried out in which the following properties were determined: flowability$\;(P_{D})$, permeability ($P_{s}^{s})$, tensile strength$\;(R_{m}^{s})$, and bending strength $(R_{g}^{s})\;$after dryingandwear resistance $\left(S_{s}^{s}\right)$, also on samples in the dry state.

Flowability$\;(P_{D})\;$was determined by the method of H.W. Dieterta and F. Valtiera [3]. It makes use of compacting molding sand with a laboratory compactor. In the test, a cylindrical sample is compacted by hitting a rammer five times. Flowability is measured by testing the loss in height of the sample measured between the fourth and fifth impact of the rammer. The value of flowability was calculated from the Formula (1):

(1) $$P_{D}=100-40x,\%$$

where:

x—difference in height of the sample between the fourth and fifth impact of the rammer [mm].

Permeability ($P_{s}^{s})$ was determined by an accelerated method on a digital apparatus designed for determining the permeability of LPiR1 (Multiserw-Morek, Marcyporęba, Poland). Determination of parameters concerning tensile strength$\;(R_{m}^{s})$ and bending strength $\left(R_{g}^{s}\right)$ was carried out on the universal testing machine LRu-2e (Multiserw-Morek, Marcyporęba, Poland). Wear resistance $\left(S_{s}^{s}\right)$ was determined according to BN-77/4024-02 standard at an ambient temperature for 3 min. The wear resistance $\left(S_{s}^{s}\right)$ was calculated from the Formula (2):

(2) $$S^{s}=\frac{a-b}{a}\;\times \;100,\;\%$$

where:

a—sample mass before testing (g),

b—sample mass after testing (g).

### 2.7. SEM Analysis of Molding Sand

In order to assess the structure of molding sand, microscopic observations were carried out by the SEM (Scanning Electron Microscope) TM 3030 (Hitachi, Tokio, Japan), cooperating with the EDX detector (Energy Dispersive X-ray Spectroscopy) TM3000 MICSF+ (Oxford Instruments, Oxford, UK).

### 2.8. Test Castings

**Figure 1:**
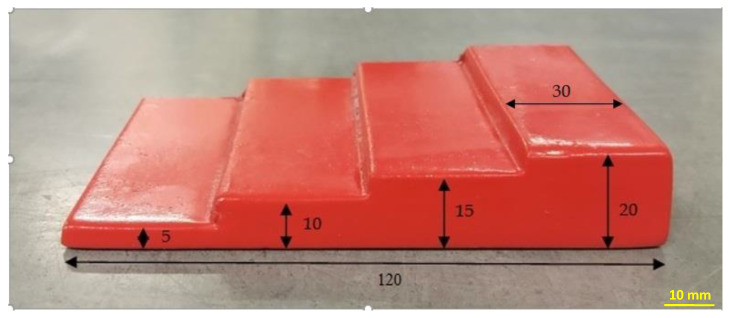
Model for molding foundry molds [27,28].

Casting molds were prepared by hand using the model shown in the photo—Figure 1, from molding sands with the addition of various binders. After obtaining the castings, we were able to assess their surface roughness depending on the molding sands used. 

The castings were made of gray cast iron EN-GJL-250. This alloy was chosen because it is a commonly used type of cast iron to evaluate the influence of molding sands on the solidification process of cast irons. Moreover, it is a typical, commonly used cast iron with good technological properties and a significant share in world production. Cast iron melts for the tests were carried out in a medium-frequency induction furnace, Type PI 30 (ELKON, Rybnik, Poland), using a crucible with a capacity of 6 kg. Gray cast iron (3.52% C; 1.80% Si; 0.76% Mn; 0.19% P; 0.01% S), ferrosilicon (FeSi75T) and a carburizer were used as charge materials.

The liquid cast iron was overheated to temperature of 1350 ÷ 1400 °C.The slag was pulled off, and carburizer and ferrosilicon in the amount of about 1.3% in relation to the weight of the cast iron were introduced. Next, liquid alloy was cast by gravity into previously prepared molds made of various molding masses. After solidification and cooling, the castings were knocked out of the molds and subjected to further tests. The chemical composition of the castings was determined using the S1 Mini Lab spectrometer (GNR Analytical Instruments Group, Milan, Italy), and the measurement results are presented in Table 2.

### 2.9. Roughness of Test Casts

**Figure 2:**
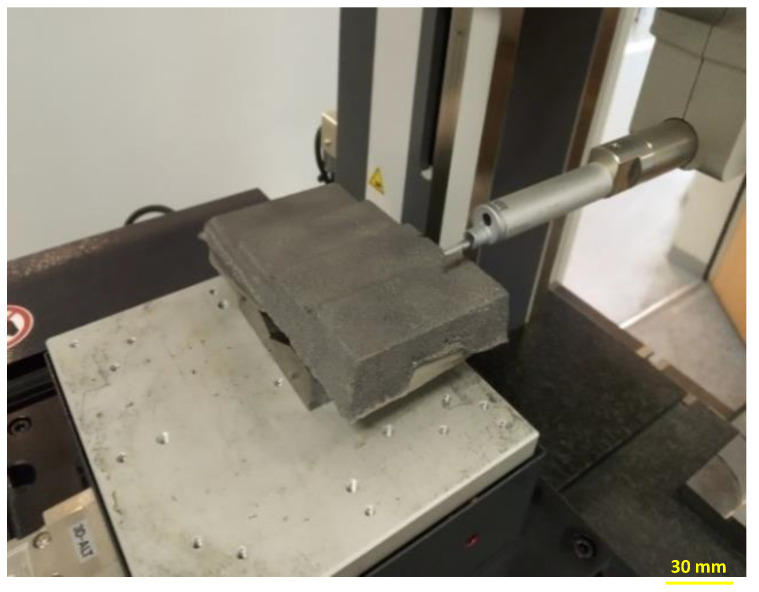
MITUTOYO CV-3200 profilometer and view of the analyzed stair cast (the roughness of each “stair” was individually measured using a needle measurement).

Measurements of surface roughness of the test castings were carried out using the SV-3200 surface roughness tester (MITUTOYO, Kawasaki, Japan)—Figure 2. The device was scaled as follows: measurement length (X) 15.0000 mm, measurement step 0.0005 mm, measurement speed 1.00 mm/s, axis range (Z) 0.800 mm. Measurements were made in accordance with EN ISO 4287: 1998/AC: 2008 and PN-EN ISO 4288: 2011.

## 3. Results and Discussions

During the pouring process, organic components of molding and core sands, mainly binders, as well as hardeners and protective coatings, usually burn quickly and evaporate, the remainder is ash [29]. During thermal decomposition of barley malt, casting defects may appear as a result of gasification. Therefore, research attempts were made to determine the content of organic compounds in barley malt and to analyze processes of thermal decomposition of the binder.

### 3.1. Tests of Barley Malt as a Binder for Molding Sand

#### 3.1.1. Determination of Starch Content

As a result of polarimetric tests, the starch content in barley malt was determined, which was 58.30%. Obtained results confirmed that starch—a carbon biopolymer composed of glucose units—is the main component of the tested binder.

#### 3.1.2. Determination of Protein Content

Using the Kjeldahl method, the total white content of binder was determined. For this purpose, the content of protein nitrogen was determined, which was 1.7312%. The amount of nitrogen was then multiplied by the nitrogen-to-protein conversion factor, appropriate for barley malt—6.25. The protein content was therefore 10.82%. 

The obtained results show that the total content of starch and protein substances constitutes nearly 70% of the ingredients contained in binder. The rest consists of the remaining sugars, including dextrins, cellulose, and fats.

#### 3.1.3. TGA/TG Analysis

**Figure 3:**
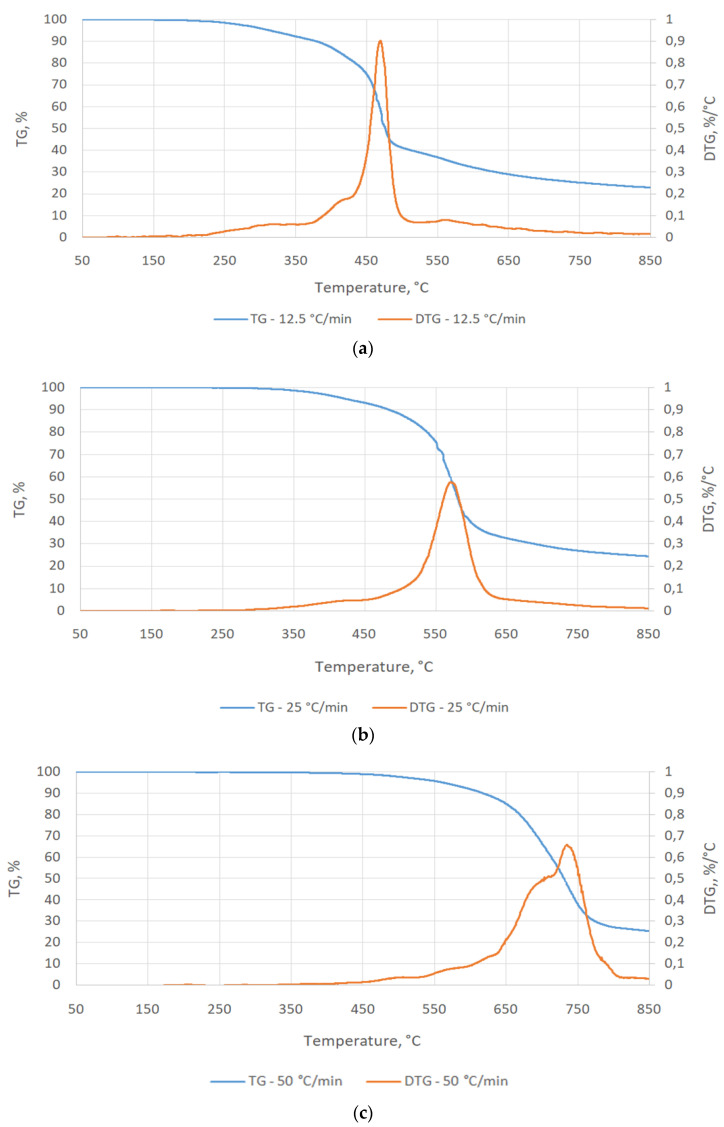
Results of TG/DTG analysis of barley malt for the heating rate: (**a**) 12.5 °C/min, (**b**) 25 °C/min, (**c**) 50 °C/min.

The results of TG and DTG tests were analyzed in order to determine the content of substances that underwent thermal decomposition. The analysis was performed by estimating the parameters of the Gauss equation for the curves visible on the graph, shown in Figure 3. Calculations were made using the OriginPro 2019b software (OriginLab, Northampton, MA, USA).

With a furnace heating rate of 12.5 °C/min, the main thermal decomposition of the sample took place when the temperature reached 440 °C and progressed to 510 °C (initial thermal degradation was 92.6 °C)—Figure 3a. At the heating rate of 25 °C/min, the weight loss of the sample could be observed in the temperature range of 500–620 °C (the initial thermal degradation was 172.0 °C)—Figure 3b, which indicates an intensive decomposition of organic matter. On heating at 50 °C/min, the weight loss of the sample could be observed when the temperature reached 650 °C (the initial thermal degradation was 218.0 °C). The decomposition of the organic matter lasted up to 800 °C—Figure 3c.

Regardless of the furnace heating rate, the tested material was characterized by a final weight of ~25% of the initial value, which was mainly ash. Thus, the loss of sample mass of ~75% was organic matter, which was mainly composed of bound carbon. This material, undergoing thermal decomposition, can cause gasification by combustion products. As is commonly known, carbon oxides (CO_2_, CO) are products of coal combustion (the main component of binder), depending on the oxygen access to the reaction system.

**Figure 4:**
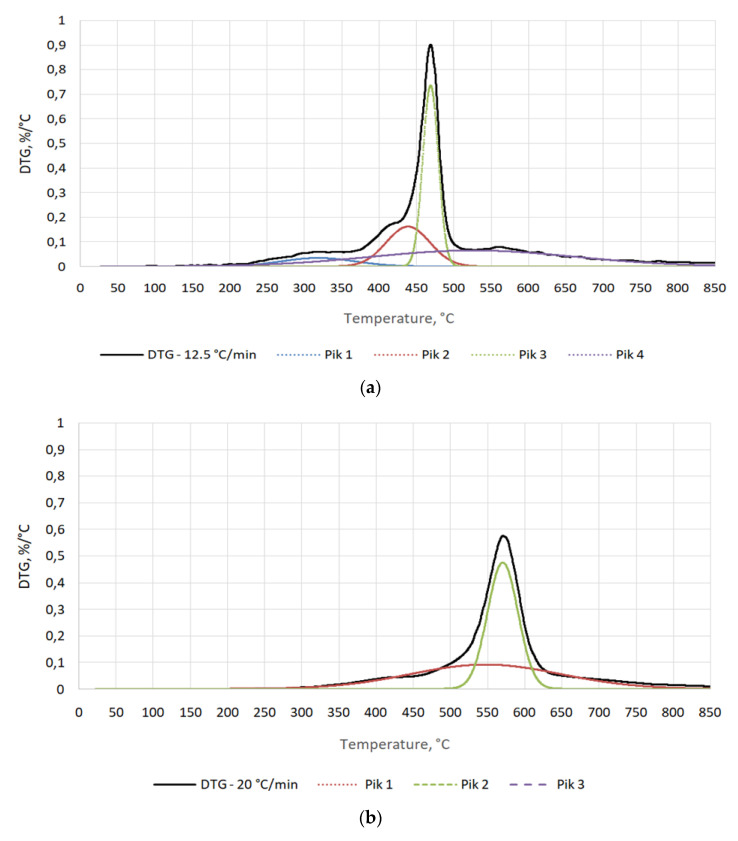

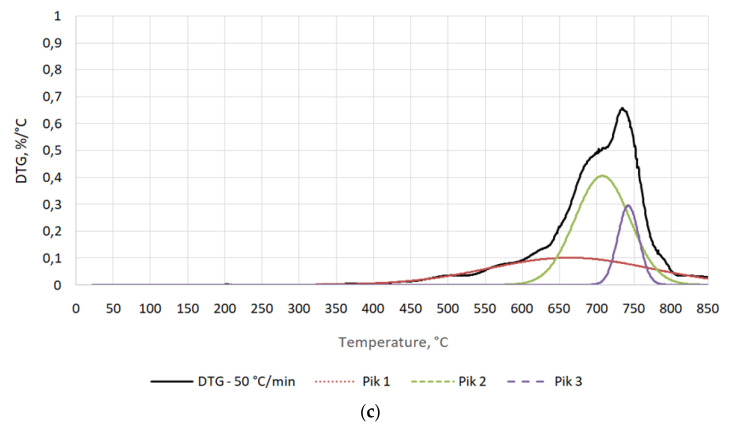
Detailed distribution of DTG diagram for furnace heating speed: (**a**) 12.5 °C/min, (**b**) 25 °C/min, (**c**) 50 °C/min.

Detailed analysis of DTG results shows that the temperature range of thermal decomposition of tested binder depends significantly on heating rate and that the tested binder may contain from 2 to 4 main compounds. These substances are thermally decomposed. This is indicated by the test results in Table 3 and Figure 4.

At a furnace heating rate of 12.5 °C/min, 4 curves appeared—Figure 4a, the maximum decomposition of which took place at temperatures of 318.2 °C, 438.7 °C, 438.9 °C, and 525.8 °C. This indicates thermal decomposition of malt organic components at these temperatures, and their content in the binder amounted to 8.5%, 20.8%, 33.8%, and 36.9%, respectively—Table 3. With the increase of the heating rate to 25 and 50 °C/min, the maxima of curves decreased, lowering and moving together toward higher temperatures. This may indicate the fact that the compounds included in the binder have similar thermal decomposition characteristics—Figure 4b,c.

Similar to the TG/DTG plot, a detailed analysis of binder thermal decomposition curves showed a significant shift in organic compound decomposition curves with an increase in heating rate. This may also be due to the large mass of the sample (2 g) used in the tests. Therefore, at a high heating rate, the tested material was not able to heat up in its entire volume. The matter distribution of the outer layers could overlap with the matter distribution of the inner layers, which reached the set value with a delay. The effect of limited heat conduction deep into the sample may be proven by the research conducted by Seruga et al. [30]. They performed a barley TGA at an oven heating rate of 10 °C/min, achieving a similar final TG ~30%, but with major malt decomposition at much lower temperatures of 200–500 °C and three decomposition curves.

### 3.2. Microscopic Evaluation of Molding Sands

In order to determine the morphology of the surface of grains and bonding bridges, microscopic observations were made using SEM with a BSE (Back-Scattered Electrons) detector. To obtain a high-resolution image, samples were carbon sprayed using a Q150T high-vacuum sputtering machine (Quorum, Laughton, UK).

**Figure 5:**
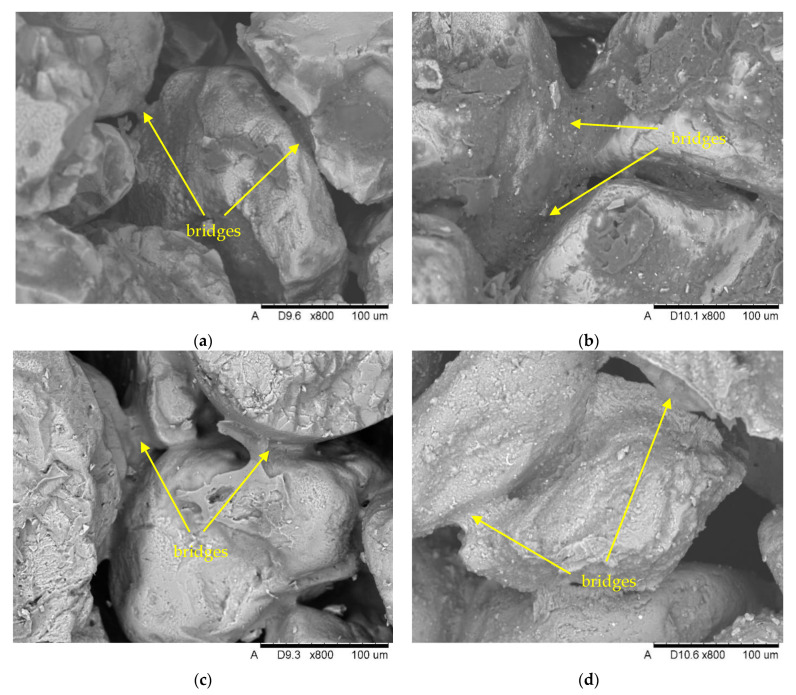

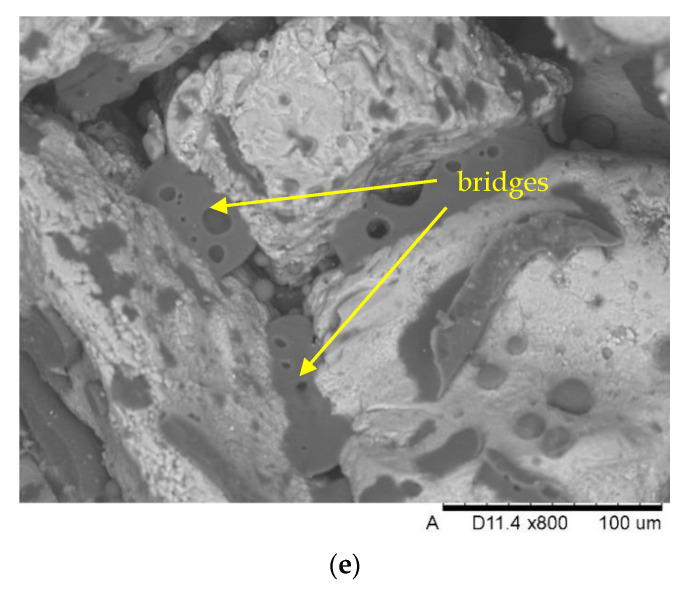
The surface morphology of molding sand for binders (SE imaging technique): (**a**) MB 2%, (**b**) MB 5%, (**c**) WG, (**d**) B 8%, (**e**) RCS.

The surface morphology of the grains and the bonding bridges for the tested masses is presented using photos in Figure 5.

The SEM image of molding sand (by SE imaging technique) with malted barley is shown in Figure 5a,b. The layer of malted barley binder creating bonding bridges between high-silica sand grains is smooth. Periodically, flakes of malted barley binder have been observed. Slowly heating the molding sand results in slow water flow into the airstream, which floats the dry load and ensures the quality of the bridges. The binder, dried in the form of smooth and mild bridges between the grains, allows very good strength properties of the molds and cores. The mass with the water glass (Figure 5c) has a very similar characteristic of bonding bridges as in the case of masses based on barley malt. However, the presence of irregularly shaped breaches was additionally noticed on the surface of the grains. The SEM image of bentonite molding sand is shown in Figure 5d. The flake structure characteristic of bentonite is visible on the grain surface. Small cracks in the bonding bridges were also observed, which translates into lower strength of these masses. Figure 5e shows topography of bridges connecting masses from sand surrounded by resin. The image shows a layer of resin evenly distributed over the matrix grains with embedded fine-dispersion spherical gas bubbles. Also, numerous “drops” of unbound binder were observed on the grain surface.

**Figure 6:**
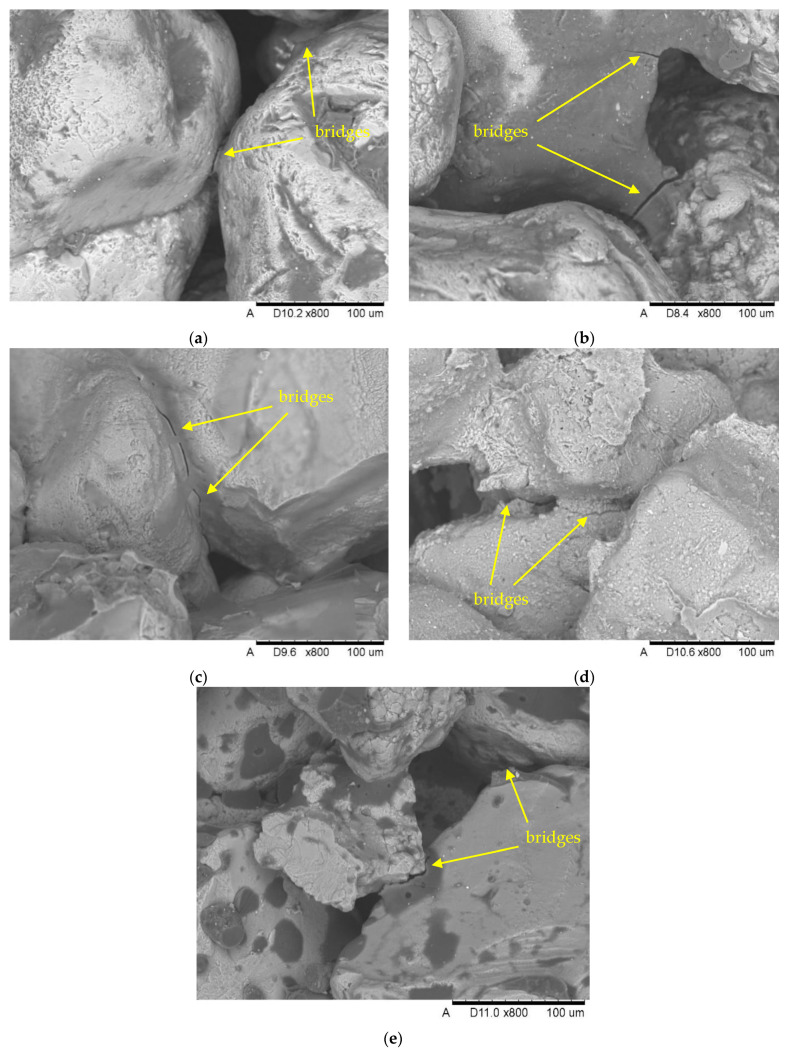
Destruction of bonding bridges after testing the bending strength of masses containing binders (SE imaging technique): (**a**) MB 2%, (**b**) MB 5%, (**c**) WG, (**d**) B 8%, (**e**) RSC.

Moreover, SEM observations of samples after testing the bending strength of masses were carried out. Figure 6 shows photos of fractures of these samples, showing destruction of bonding bridges.

Masses based on barley malt (Figure 6a,b) and a water glass (Figure 6c) are characterized by the destruction of the cohesive type, i.e., the system in which the adhesive forces are greater than the cohesive forces [31,32]. The lower strength of the molding mass with the addition of 2% of barley malt binder compared to 5% is due to the so-called droplet distribution of binder, which is characteristic of the lower binder content and its high viscosity. The SEM image of bentonite mass (Figure 6d) is an example in which forces of adhesion and cohesion balance each other, and therefore destruction takes place on the grain surface of sand matrix and inside the layer of binding material [31]. In the case of a sand mass surrounded by resin (Figure 6e), there is a typical adhesive destruction.

### 3.3. Properties of Molding Sand

**Figure 7:**
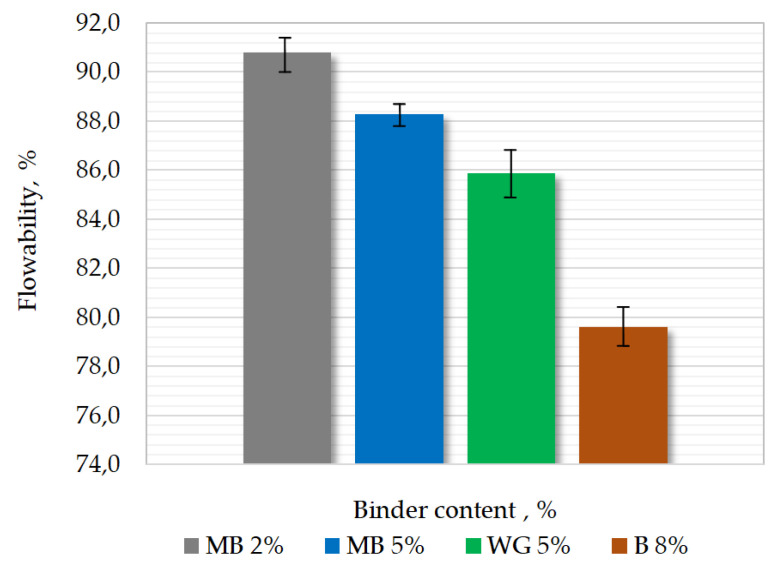
Results of flowability measurements of molding sands with different binders.

The results of the flowability$\;P_{D\;}$of masses are shown in Figure 7. Comparing this with the results for binder from barley malt, there is a noticeable tendency toward a decrease in flowability with an increase in the binder content. If a mass of B 8% is added to this conclusion, the above-mentioned trend is confirmed. Comparing the obtained results, it can be seen that all masses obtained good flowability, because the flowability exceeded or was close to 80%. It allows us to conclude that the tested masses can be compacted by all known methods, from manual forming to various machine forming methods.

Increasing the binder content, resulting in a reduction in flowability, is related to a significant difference in the size of sand and binder grains, resulting in an increase in the value of the internal friction coefficient [33].

**Figure 8:**
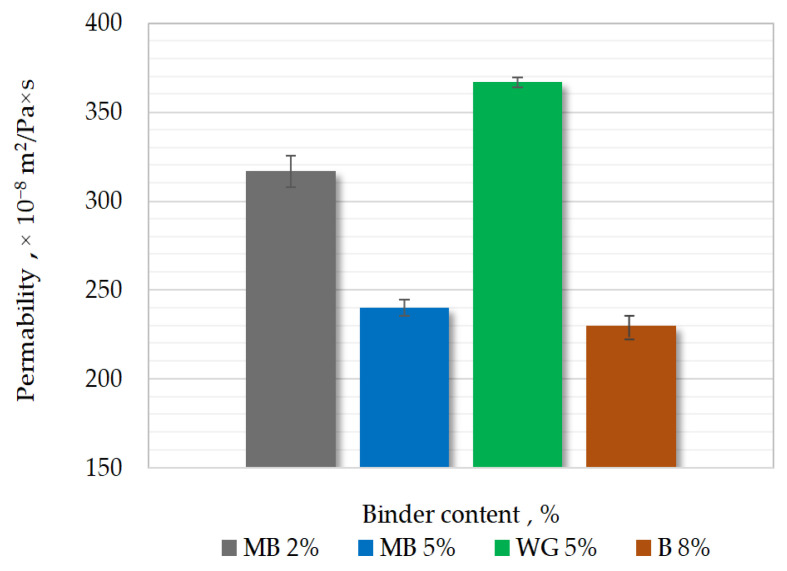
Results of permeability measurements of different molding sands.

Results of the permeability $P_{s}^{s}$ of molding sand are shown in Figure 8. A trend was observed that with increasing binder content, the permeability decreased. This is related to the greater number of voids between sand grains in the masses with a lower binding material content [6,8,9].

During pouring with liquid metal, significant amounts of gases can be released from molds with organic binders. Therefore, a lower malt content (2%) is preferred.

**Figure 9:**
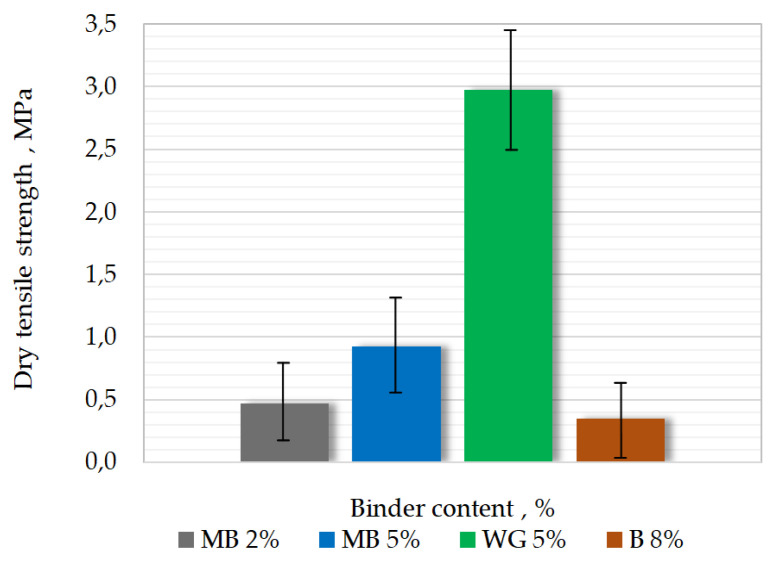
Results of dry tensile strength measurements of different molding sands.

The results of $R_{m}^{s}$ from the tensile test are shown in Figure 9. Increasing the binder content in MB masses resulted in a significant increase in tensile strength. The strength $R_{m}^{s}$ of the mass with the content of 5% of barley malt was highest.

**Figure 10:**
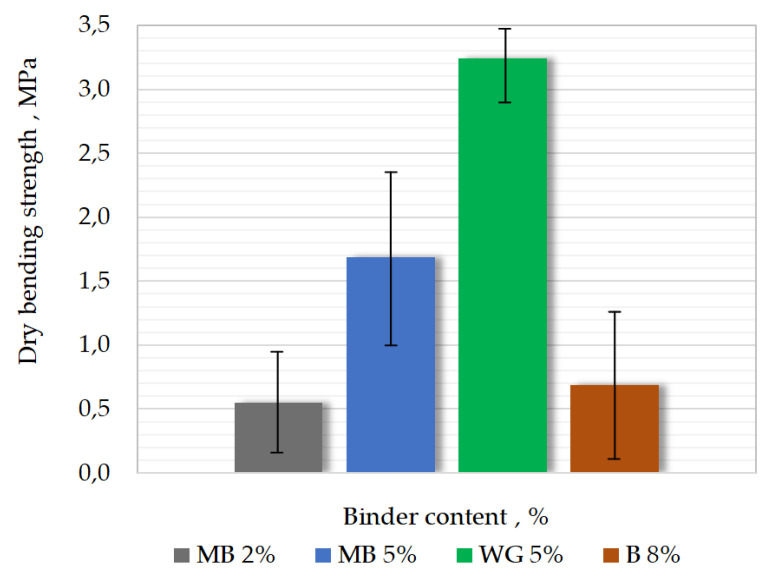
Results of dry bending strength measurements of different molding sands.

Results of measurements of bending strength$\;R_{g}^{s}$ of masses are shown in Figure 10. It can be seen, as with the tensile strength results, that increasing binder content increases the bending strength of the mass with malt binder. According to data from the manufacturer, bending strength for molding sand made of coated sand is close to 8 MPa. The manufacturer does not carry out other tests that were performed in this work.

**Figure 11:**
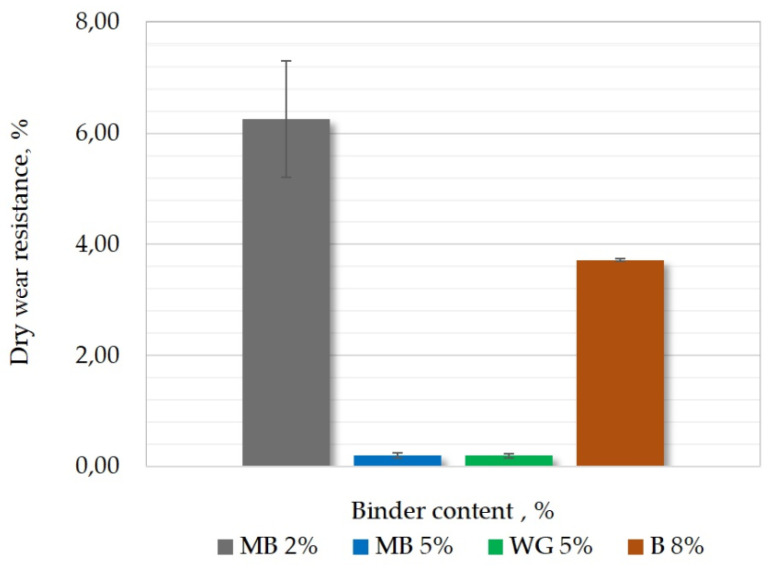
Results of dry wear resistance measurements of different molding sands.

Figure 11 shows the results of wear resistance $S_{s}^{s}$. Increasing binder content reduces weight loss during the measurement. Reducing the value of $S_{s}^{s}$ may mean a smaller number of raw casting defects.

### 3.4. Macroscopic Evaluation of Test Castings

#### 3.4.1. Visual Assessment

**Figure 12:**
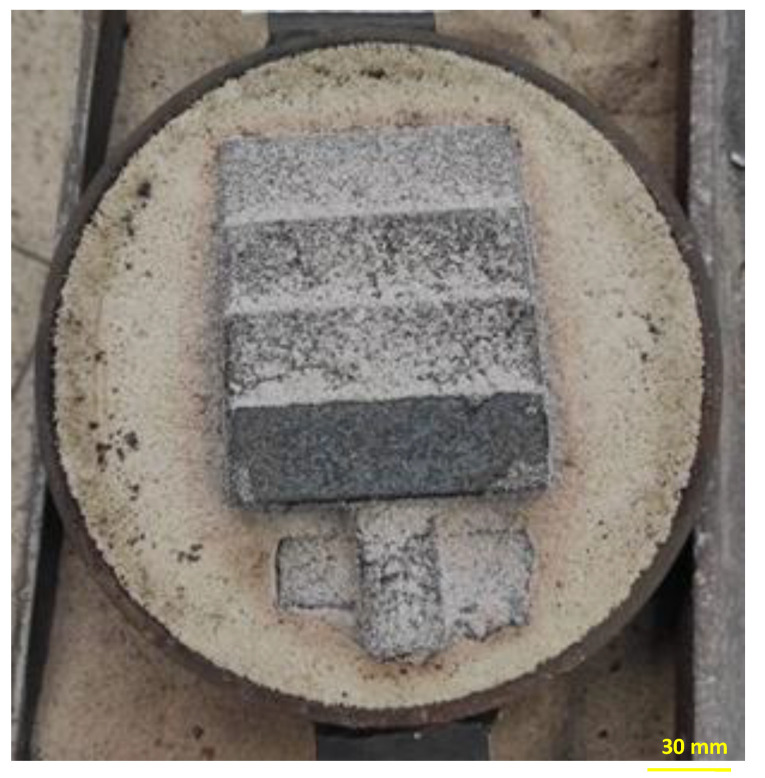
Open form after cooling, made of a mass containing 2% MB.

Preliminary visual analysis showed that the casting process was carried out correctly and all the obtained castings showed no signs of damage. Figure 12 shows a photo of the crude casting obtained in a mold made of a MB 2% mass (it is confirmed by a correctly conducted casting process).

The thickness of the burnt layer of MB 2% and MB 5% was also assessed. A clear difference in the thickness of the burnt molding sand layer was observed. In the case of MB 2%, the thickness was ~14.5 mm and MB 5%~20 mm.This difference is related to higher malt content in the 5% MB mass, which translates into a greater depth of the scorch zone. In mass with a binder content of 2%, a greater amount of quartz sand eliminates the possibility of such a deep combustion of malt during the casting process.

#### 3.4.2. Surface Roughness of the Castings

In order to determine the surface roughness, castings were sandblasted. Even a preliminary visual assessment showed differences in roughness between individual castings. Roughness measurements were made along each “stair” from 1 to 4 of all the test casts. Values of Ra—arithmetic mean deviation of profile from the mean line and value of Rz—sum of height of highest elevation and deepest depression were determined. Averaged measurement results are presented in Table 4.

**Figure 13:**
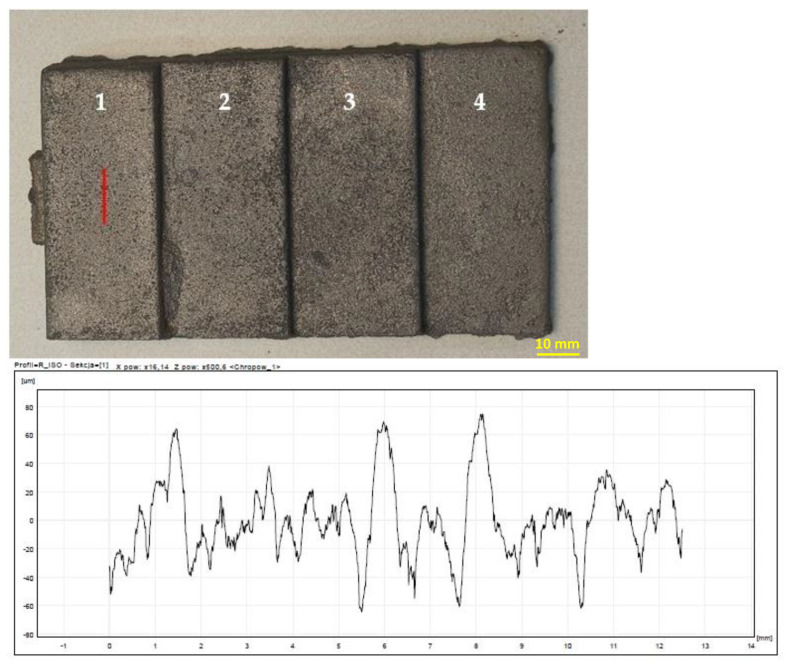
Raw casting made in a mold with a mass of B 8%. A chart of an example of surface roughness for the 1st “stair” of the casting. 1–4 are numbers of stairs of the casting.

**Figure 14:**
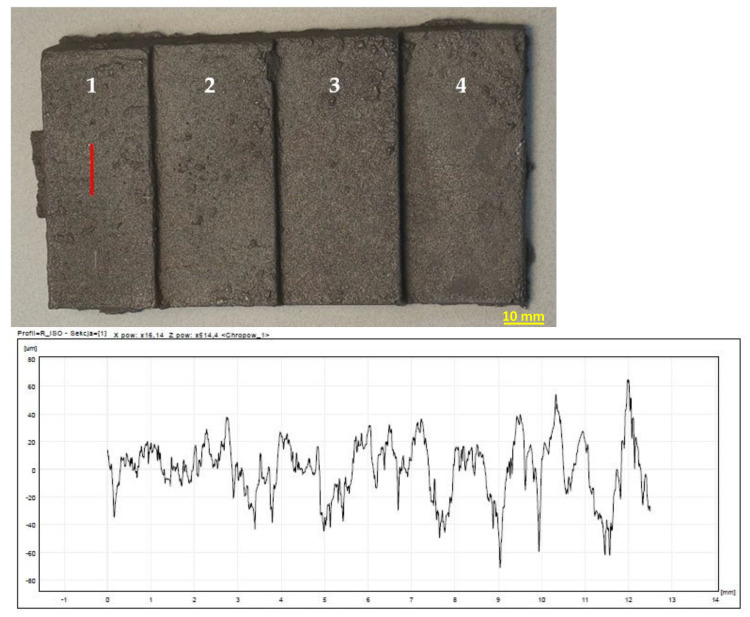
Raw casting made in a mold with a mass of RCS. A chart of an example of surface roughness for the 1st “stair” of the casting. 1–4 are numbers of stairs of the casting.

**Figure 15:**
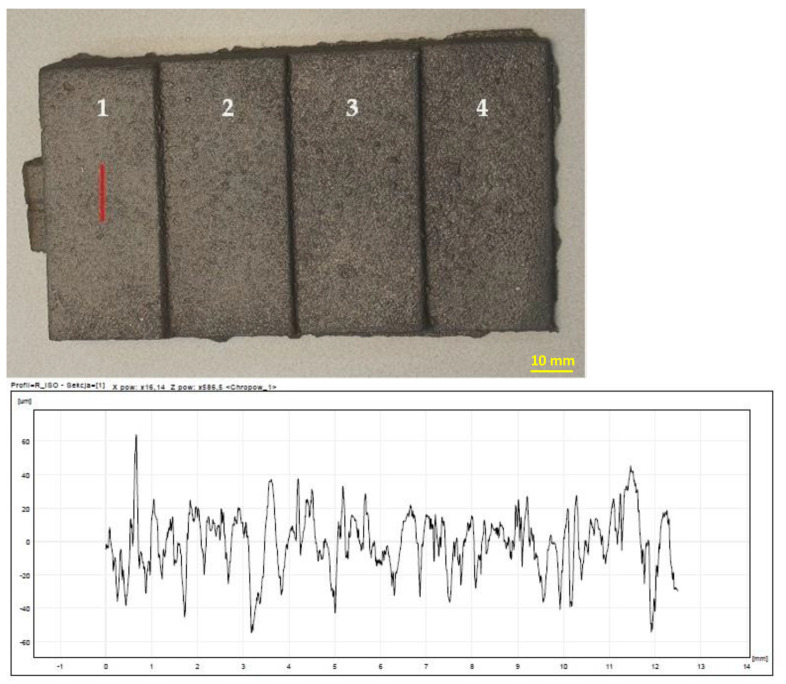
Raw casting made in a mold with a mass of MB 2%. A chart of an example of surface roughness for the 1st “stair” of the casting. 1–4 are numbers of stairs of the casting.

**Figure 16:**
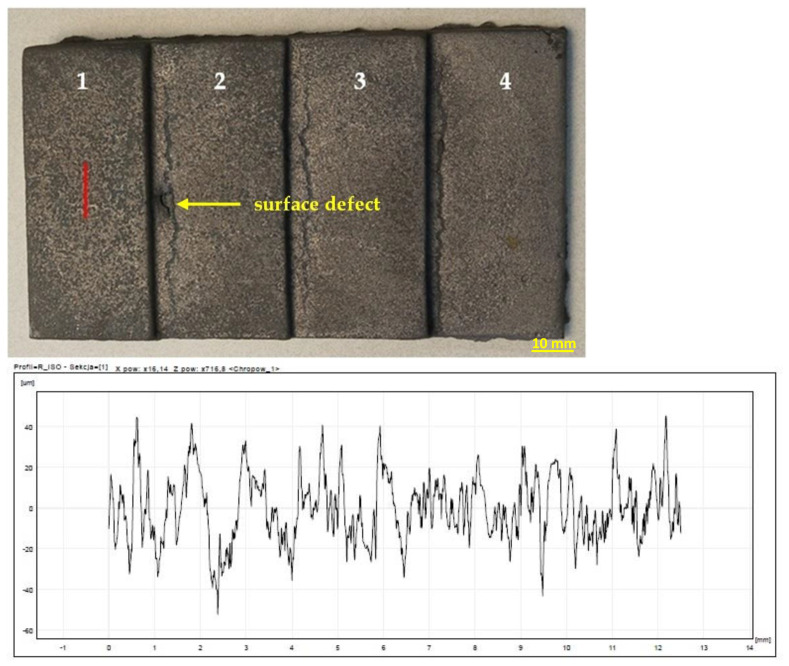
Raw casting made in a mold with a mass of MB 5%. A chart of an example of surface roughness for the 1st “stair” of the casting. 1–4 are numbers of stairs of the casting.

**Figure 17:**
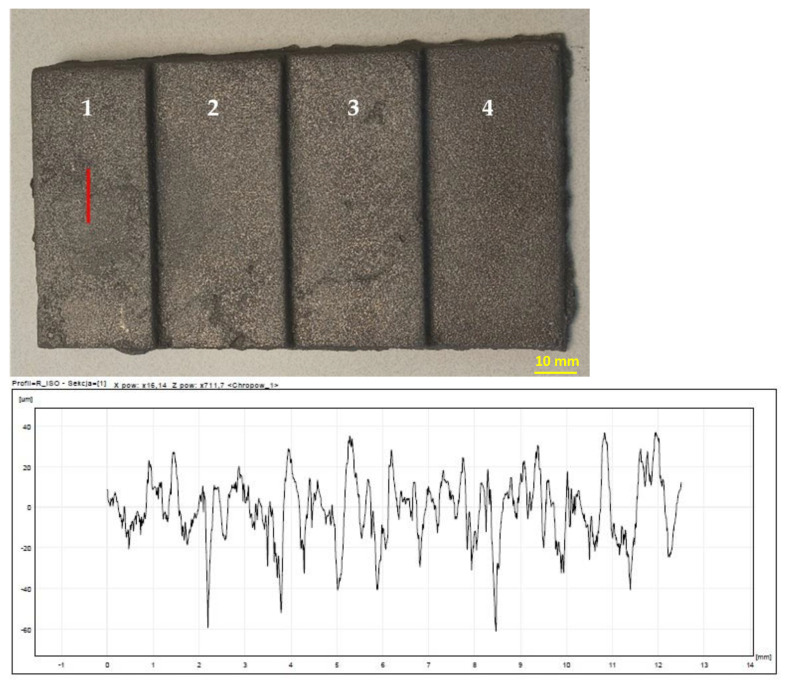
Raw casting made in a mold with a mass of WG 5%. A chart of an example of surface roughness for the 1st “stair” of the casting. 1–4 are numbers of stairs of the casting.

The highest roughness was observed on the surface of the casting obtained in the mass of B 8%—Figure 13. In turn, the lowest roughness was characteristic of the cast made of RCS mass—Figure 14. The results of the roughness measurements of castings cast into molds from masses containing MB in the amount of 2% (Figure 15) and 5% (Figure 16) turned out to be satisfactory and comparable with results obtained for casting made in sand molds with WG (Figure 17). This applied in particular to casting made in a mold based on mass with the addition of 5% MB. In this case, surface defects in the form of cracks were observed on the surface of “stair” (Figure 16). The relatively high content of barley malt in the mass meant that during the solidification of the liquid metal, the binder burned intensively and a large amount of gases was released. The gasification probably caused the appearance of defects on the surface of solidified casting.

## 4. Conclusions

The addition of MB binder in the amount of both 2% and 5% to mass guarantees relatively good strength properties, required from commonly used molding sands. Increasing the content of this binder increases the strength of the mass. It is related to the formation of bonding bridges between the binder and the molding sand, similar to the case of sand with a water glass.

High flowability allows us to draw the conclusion that molding sand with MB binder can be compacted by standard methods. The mass containing MB 5% was characterized by lower fluidity compared to the mass with an MB 2% addition. This is due to the higher content of sticky material, resulting in a higher viscosity.

The results of the TG/DTG analysis show that the loss of ~75% of the mass of the MB binder is organic matter. Over 50% of this is starch, which, undergoing thermal decomposition and gasification, may cause surface defects in castings. This dependence was observed for the mass containing 5% MB. There were no such defects in the case of the MB 2% mass. Thus, it is possible to eliminate this problem by introducing a smaller amount of binder into the molding sand or by using appropriate venting solutions. In addition, the greater content of binder in the mass resulted in its lower permeability. Gas activity of the binder will be the subject of further studies.

Moreover, the mass of MB 2% was characterized by formation of a burnt layer 25% smaller than the mass of MB 5%. It is extremely important from the point of view of regeneration of the molding sand. This is due to the exothermic reaction when more binder is burnt.

On the basis of the obtained results, it can be concluded that barley malt (compared to reference binders) can be a binder for molding sand. In addition, it is a natural resource, renewable through agricultural production. Therefore, in relation to conventional binding materials, it can be an alternative material that fits into the concept of sustainable development.

## Figures and Tables

**Table 1:** Composition of molding sands used in the research.

Fine Silica Sand (%_mass_)	Binder Type	Binder (%_mass_)	Distilled Water (%_mass_)
98	barley malt—MB 2%	2	2
95	barley malt—MB 5%	5	5
95	water glass—WG	5	-
92	bentonite—B 8%	8	0.8
97	resin coated sand—RCS	3	-

**Table 2:** Chemical composition of the cast iron used for the test castings.

	Chemical Composition [%_mass_]										
Cast iron	C	Si	Mn	Ni	Cu	Mo	Cr	Al	P	S	Fe
	3.55	3.32	0.46	0.14	0.20	0.11	0.03	0.03	1.21	0.20	rest

**Table 3:** Characteristic values of DTG curves for different heating rates.

DTG, °C/min	Characteristic Values	Curve 1	Curve 2	Curve 3	Curve 4
12.5	T_max_, °C	318.2	438.7	468.9	525.8
	Share, %	8.5	20.8	33.8	36.9
25	T_max_, °C	547.4	570.5	-	-
	Share, %	49.4	50.6	-	-
50	T_max_, °C	663.3	707.8	742.6	-
	Shar, %	35.4	48.1	16.4	-

**Table 4:** Results of roughness measurements of castings made of molds from molding sands with the addition of various binders.

Measurement for an Individual “Stair” of Test Castings		Roughness Parameters for Castings Made in Molds from Masses with Various Binders				
		MB 2%	MB 5%	WG 5%	B 8%	RCS
1	Ra_av_	10.8130	8.2973	12.6998	18.8292	8.1663
	Rz_av_	71.8486	58.5037	69.0820	87.2708	83.5917
2	Ra_av_	13.8019	12.9511	14.8688	22.4032	6.4821
	Rz_av_	79.7763	79.4316	81.4624	104.0351	92.6132
3	Ra_av_	13.9996	16.004	12.3565	18.1374	10.633
	Rz_av_	89.3288	90.9578	81.4827	92.1897	89.5778
4	Ra_av_	11.5767	14.5426	11.8558	20.3440	10.5243
	Rz_av_	79.5225	90.9488	75.8473	102.5992	86.0159
Average value from all “stair”	Ra_av_	12.55	12.95	12.95	21.43	8.95
	Rz_av_	80.12	79.96	76.97	96.52	87.95

## Data Availability

Not applicable.
